# The use of a pre-operative scoring system for the prediction of phacoemulsification case difficulty and the selection of appropriate cases to be performed by trainees

**DOI:** 10.1186/1471-2415-6-38

**Published:** 2006-12-27

**Authors:** Stuart A Osborne, Phillip Severn, Catey V Bunce, Scott G Fraser

**Affiliations:** 1 Sunderland Eye Infirmary, Queen Alexandra Road, Sunderland, SR2 9HP, UK; 2 Research and Development, Moorfields Eye Hospital, 162 City Road, London EC1V 2PD, UK

## Abstract

**Background:**

To establish whether a previously validated scoring system (Habib) for the prediction of risk or likelihood of posterior capsule rupture during phacoemulsification surgery could be used to: 1. Predict the difficulty of a phacoemulsification case, and 2. Select appropriate phacoemulsification cases for trainees.

**Methods:**

The study sample was consecutive phacoemulsification cases undertaken by senior surgeons at a single ophthalmic unit over a three-week period (170 cases). Each case was scored using a potential difficulty scoring system. Immediately post-operatively, each case was given two scores by the operating surgeon (who was masked with regard to the potential complication score). The first score indicated the perceived difficulty of the case, and the second score, the degree of experience that they thought a trainee would require in order to have performed the same case without complication.

**Results:**

Using Cuzick's non-parametric test for trend, there was evidence for a trend of increasing perceived difficulty with increasing potential difficulty score (p = 0.05), and of increasing experience required with increasing potential difficulty score (p < 0.001)

**Conclusion:**

The authors advocate that Habib's potential difficulty scoring system can be used to inform the surgeon of the likely difficulty of a phacoemulsification case and to aid selection of appropriate cases for trainees prior to surgery.

## Background

Until now, prediction of the expected *difficulty *of a phacoemulsification procedure has been based on a pre-operative *subjective *assessment of the patient by the operating surgeon.

An *objective *system to predict posterior capsule rupture during phacoemulsification surgery has been formulated by Habib *et al *('potential difficulty score'), and has been validated by the authors [1,2]. Although this scoring system was devised to predict the potential *difficulty *of an individual case, until now it has been shown only to be predictive of *complication*, i.e. posterior capsule rupture, but not case *difficulty*. Whilst it is likely that a difficult case is more likely to be complicated, many difficult cases are completed without the occurrence of a complication.

Knowledge of the likely difficulty of a particular case would be useful for the selection of appropriate cases for trainee surgeons, based on their surgical experience. It could also have implications with regard to obtaining patients' consent for surgery, and could allow the fairer comparison of operating times and surgical outcomes in surgeons with differing case-mix difficulty.

The aim of this study was to establish whether this scoring system could be used to:

1. Predict the potential *difficulty *of a case; and

2. Select appropriate cases to be performed by trainees based on their previous phacoemulsification experience.

## Methods

The study sample was consecutive patients undergoing phacoemulsification surgery by senior surgeonsat a single ophthalmic unit over a three-week period. During this period, each case was given a points score using a scoring system devised by Habib et al (this was scored by SO and PS) [1]. The scoring system uses data fromthe patient'spre-operative notes, and is based on the principle of allocating points for individual risk factors thought to increase the likelihood of complications during surgery. The points are then summated to provide an overall score for each case pre-operatively i.e. a 'potential difficulty score'. Note that although this is a preoperative assessment system the surgeons were asked to complete it **post-operatively**. The points allocated to each risk factor using each system are shown in Table 1.

**Table 1:** Points allocated to each risk factor using Habib's potential difficulty score system.

**Risk factor**	**Points allocated**
**Unable to lie flat (spinal deformity, asthma, heart failure)**	1
**Severe anxiety**	1
**Head tremor**	1
**Previous angle closure glaucoma**	1
**History of complication in fellow eye**	1
**Previous vitrectomy**	1
**Corneal scarring/cloudiness**	1
**Shallow anterior chamber**	1
**Poor pupillary dilation and/or posterior synechiae**	1
**Pseudoexfoliation**	1
**Phacodonesis/weak zonules**	1
**High myopia (axial length >27 mm)**	1
**High hypermetropia (axial length <20 mm)**	1
**Nuclear density grade 1–2**	1
**Nuclear density grade 3**	2
**Mature/brunescent/white/dense/total cataract**	3

**Figure 1:**
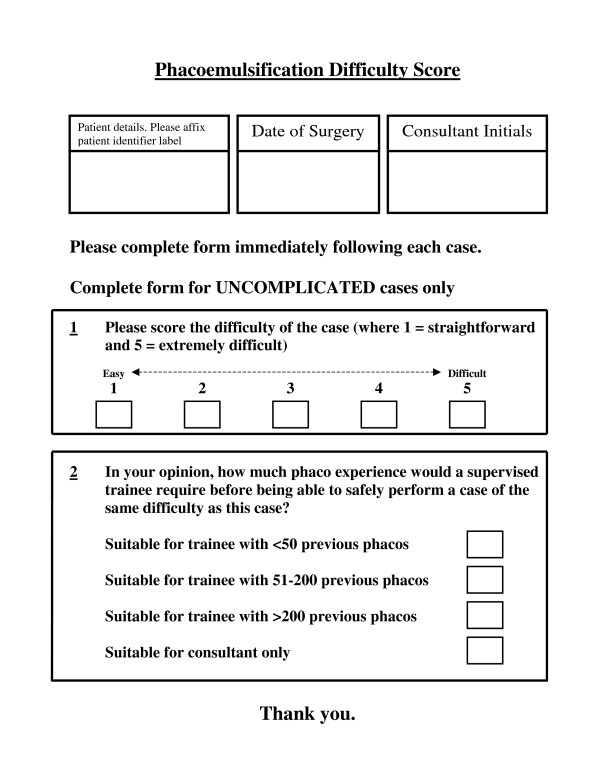
Questionnaire to operating surgeon (Completed immediately following each case).

Immediately post-operatively, each case was given two scores by the operating surgeon on a printed questionnaire (Figure 1). Though the operating surgeon was unaware of the potential difficulty score for the case, they were privy to all information in the patients' notes and would have been aware of any potential complicating factors for each case.

The surgeons were asked to complete questionnaires only for uncomplicated cases, as it was felt that cases in which a complication actually occurred would naturally be scored highly in terms of both their difficulty and their suitability for trainees.

All of the questionnaires were collated at the end of the study period in order that the data could be examined.

Ethics approval for this study was obtained from Sunderland Local Research Ethics Committee on 24^th ^January 2005. REC reference number: 05/Q0904/2.

## Results

170 phacoemulsification consecutive operations were included cases in this study.

The relationships between the allocated potential complication score and the surgeons' scores of perceived difficulty and recommended trainee experience are show in Table 2.

**Table 2:** The relationships between the allocated potential complication score and the surgeons' scores of perceived difficulty and recommended trainee experience.

		**Surgeon's perceived difficulty score**						**Surgeon's assessment of recommended trainee experience**				
		
		*1*	*2*	*3*	*4*	*5*	*Total*	*<50*	*51–200*	*>200*	*Consultant only*	*Total*
**Pre-operative potential complication score**	**1**	66	42	16	0	1	**125**	43	46	28	8	**125**
	**2**	18	8	8	2	4	**40**	7	13	15	5	**40**
	**3**	3	0	0	0	1	**4**	1	1	0	2	**4**
	**4**	0	0	0	1	0	**1**	0	0	1	0	**1**

**Total**		87	50	24	3	6	**170**	51	60	44	15	**170**

Using Cuzick's non-parametric test for trend, there was evidence for a trend of increasing perceived difficulty with increasing potential complication score (p = 0.05).

Using the same test, there was evidence for a trend of increasing experience required with increasing potential complication score (p < 0.001)

## Discussion

Our results would suggest that Habib's 'potential difficulty score' can, indeed, be used to inform the surgeon of the likely *difficulty *of a phacoemulsification case as well as the likelihood of a posterior capsule rupture [2]. This has important implications in terms of the selection of cases to be performed by trainees, obtaining consent from patients, and comparing outcomes from surgeons with differing case-mix difficulty.

The application of such a system in practice would allow the selection of a case of appropriate potential difficulty for a trainee based on that trainee's previous phacoemulsification experience. This would, in theory, allow trainees to be exposed to cases of increasing difficulty in a more controlled and graduated manner, thereby potentially reducing rates of complication.

The apparent correlation of the potential difficulty score with the actual difficulty of a case also has implications relating to the pre-operative counselling of patients. Patients with higher scores could be informed that their case, as well as having a higher risk of complication, is likely to be more difficult, and therefore may take a longer time to perform than a case with a lower potential difficulty score. This could affect the patient's decision regarding the choice of anaesthesia, and possibly their decision whether or not to proceed with surgery, particularly if they suffer from breathing difficulties, neck problems or claustrophobia.

One further application of the potential difficulty score would be in the area of surgical audit and the revalidation of surgeons. Such a scoring system could allow a more unbiased comparison of the duration of surgery and surgical outcome for surgeons with different case mix difficulty.

Habib et al designed their system based on a questionnaire to ophthalmic consultants in which risk factors predisposing to intraoperative complication were ranked. They also used information from previous work by Willerscheidt *et al *[3] and Najjar and Awwad [4].

This has resulted in the formulation of a system predictive of operative difficulty by the use of information which is readily available from the pre-operative notes and/or pre-operative assessment of the patient. The ease of use and speed of application of this system is an important practical consideration if it is to be employed in clinical practice.

Future work is required to establish the significance of *individual *risk factors by a more objective means, and allocate an appropriately weighted score for each risk factor accordingly. This, however, will require a very large scale study.

As we discussed in our previous study, Habib's system does not credit posterior polar cataracts or traumatic cataracts (without clinical evidence of zonular weakness) as risk factors, despite the fact that these factors can be associated with a higher risk of surgical complication [2,5-8].

We would recommend that cases with posterior polar cataract and cases where there is a history of significant ocular trauma, with significantly more advanced cataract in the traumatised eye (even where there is absence of clinical evidence of zonular weakness/dehiscence) should be undertaken with caution.

We acknowledge that there were cases in our study where the potential complication score and the operating surgeon's assessment of the case difficulty did not correlate closely. Of particular interest were cases in which the potential complication score was low, but the surgeon's case difficulty score was high. The surgical notes of such cases were examined, and in most cases, a reason for the difficulty was not stated or the cause was unpredictable from the pre-operative patient assessment (e.g. patient coughing during surgery, patient movement during surgery, poor eye position, etc.).

This reflects the fact that a scoring system such as this is not flawless in terms of its predictive power, and that there will always be surgical surprises (albeit, sometimes pleasant ones) relating to case difficulty.

We also acknowledge a potential bias of the study, in that the operating surgeon who was scoring a case in terms of its difficulty had access to the patient and their notes pre-operatively and, therefore, would be aware of potential causes of difficulty during the surgery. Whilst, for the purposes of validating the scoring system, it would have been desirable for the operating surgeon to be masked with regard to a patient's pre-operative status, this was not ethical.

For this reason, the surgeon was asked to score each case immediately following the surgery (and not at the end of the operating session) in the hope of obtaining a true impression of the difficulty of each case. Another potential flaw with our study was the fact that different surgeons may have differing opinions as to the surgical experience required by a trainee in order to perform a case safely. For this reason, as many surgeons as possible were asked to participate in the study in the hope of gaining a consensus of opinion regarding the trainee experience required. In total, seven different senior surgeons completed assessment forms for their cases during the study period. This represents the majority of phacoemulsification surgeons in our unit, and the authors are of the opinion that should provide a reasonable consensus of opinion. We decided to exclude those cases that did have complications as we felt it unlikely that the scoring surgeons would rate a complicated case as technically easy i.e. there would be an understandable loss of objectivity when scoring these complicated cases.

Despite these potential biases and flaws, Habib's potential difficulty scoring system does, indeed, appear to be of help in predicting the difficulty of phacoemulsification cases, and does correlate with senior grade surgeons' consensus of opinion with regard to the trainee experience required to perform such cases with reasonable safety.

As such, the authors would advocate that this scoring system could be used to aid the selection of appropriate cases for trainees of varying experience.

The authorsrecommend that:

• Cases scoring 1 with Habib's system are suitable for all trainees;

• Cases scoring 2 should be performed only by traineeswho have performedmore than 50 previous phacoemulsifications, and;

• Cases scoring 3 or more should be performed only by trainees who have performed at least 200 previous phacoemulsifications or by senior grade surgeons.

## Conclusion

The authors advocate that Habib's potential difficulty score can be used to help inform the surgeon of the likely *difficulty *of a phacoemulsification case, and that itcan be used to aid the selection of appropriate cases for trainees of varying experience.

This scoring system is also of value in obtaining informed consent of patients undergoing phacoemulsification surgery, in terms of imparting to the patient both the likely difficulty of the case, and the risk of complication.

This system also has practical applications for the audit of surgical outcomes from surgeons with differing case mix difficulty, and in the revalidation of surgeons.

Further studies would be of value in order to refine this scoring system by establishing the influence of individual risk factors on surgical outcome. These would require a much a larger study than our current one but could be used not only to check our results but to look at some of these more subtle influences on the rate of complications.

## Competing interests

The author(s) declare that they have no competing interests.

## Authors' contributions

SO and SF conceived the study. SO and PS conducted the study. CB analysed the study. All authors contributed to the writing of the manuscript and all read and approved the final manuscript.

## Pre-publication history

The pre-publication history for this paper can be accessed here:


